# Determination of *Medicago orbicularis* Antioxidant, Antihemolytic, and Anti-Cancerous Activities and Its Augmentation of Cisplatin-Induced Cytotoxicity in A549 Lung Cancer Cells

**DOI:** 10.3390/plants13030442

**Published:** 2024-02-02

**Authors:** Abdullah A. Shaito, Islam Omairi, Najlaa Al-Thani, Fatiha Seglab, Esraa Ad-Darwish, Firas Kobeissy, Salam Nasreddine

**Affiliations:** 1 Biomedical Research Center, Qatar University, Doha P.O. Box 2713, Qatar; 2 College of Medicine and Department of Biomedical Sciences at College of Health Sciences, Qatar University, Doha P.O. Box 2713, Qatar; 3 Department of Biological and Chemical Sciences, Faculty of Arts and Sciences, Lebanese International University, Beirut 1105, Lebanon; 4 Biology Department, Faculty of Sciences-Section I, Group of Anti-Cancer Therapeutic Approaches (ATAC), Laboratory Rammal Rammal, Lebanese University, Beirut 1102, Lebanon; 5 Research and Development Department, Barzan Holdings, Doha P.O. Box 7178, Qatar; 6 Biotechnology in Forensic Science Program, Faculty of Health Sciences, American University of Science and Technology, Beirut 1100, Lebanon; 7 Department of Neurobiology, Center for Neurotrauma, Multiomics & Biomarkers (CNMB), More-House School of Medicine, 720 Westview Dr. SW, Atlanta, GA 30310, USA; 8 Doctoral School of Science and Technology, Research Platform for Environmental Science (PRASE), Lebanese University, Beirut 1102, Lebanon

**Keywords:** *Medicago orbicularis*, lung cancer, herbal medicine, antioxidants, hemolytic, cisplatin, A549 cells

## Abstract

The anti-lung cancer properties of the plant *Medicago orbicularis* have not been explored yet. Therefore, we identified its phytochemical composition and investigated the antioxidant, anti-hemolytic, and anti-cancerous properties of extracts of this plant in A549 human lung adenocarcinoma cells. The results show that all parts of *M. orbicularis* (stems, leaves, and fruits) exhibit remarkable hemolytic activities and modest antioxidant capacity. In addition, all extracts showed a dose-dependent anti-cancerous cytotoxic activity against A549 cells, with fruit extracts being the most potent. This cytotoxic effect could be related, at least partly, to the induction of apoptosis, where *M. orbicularis* fruit extracts reduced the ratio of anti-apoptotic BCL-2/pro-apoptotic BAX, thereby promoting cellular death. Furthermore, the use of *M. orbicularis*, in combination with a conventional chemotherapeutic agent, cisplatin, was assessed. Indeed, the combination of cisplatin and *M. orbicularis* fruit extracts was more cytotoxic and induced more aggregation of A549 cells than either treatment alone. GC-MS analysis and total polyphenol and flavonoid content determination indicated that *M. orbicularis* is rich in compounds that have anti-cancerous effects. We propose *M. orbicularis* as a potential source of anti-cancerous agents to manage the progression of lung cancer and its resistance to therapy.

## 1. Introduction

Cancer remains the second leading cause of disability and death worldwide, where around one in six deaths are related to cancer. In 2020, around 19 million new cancer cases and almost 10 million cancer-related deaths were recorded globally [1].

Lung cancer has a high incidence rate; it was the second most commonly diagnosed cancer in 2020. Lung cancer is the leading cause of cancer-related deaths (around 1.8 million deaths or 18% of global cancer-related deaths in 2020) [1]. With a 21% 5-year survival rate, lung cancer has one of the most poor prognoses [2]. A significant obstacle in lung cancer therapy is the resistance that it develops to chemo- and radiotherapy. Following therapy, resistance can lead to the relapse of lung cancer around 6 months post-therapy in many patients [3,4]. Cisplatin is a cytotoxic chemotherapeutic agent used to treat several cancers including lung cancer [5]. The standard of treatment for limited-stage lung cancer remains chemotherapy, using four-to-six cycles of cisplatin and etoposide and concurrent radiation therapy. However, 60–70% of patients are at extensive-stage lung cancer and require a different treatment regimen, also involving cisplatin [4,6]. Nevertheless, these treatments can become refractory in patients. When relapse takes place, lung cancer is usually refractory to treatment and has poor prognosis due to the limited availability of therapeutic options [3,4]. As a result, there is an urgent demand for new treatment options and alternative therapeutic modalities that can treat lung cancer and circumvent its chemoresistance. Herbal medicine represents a promising alternative in this regard.

Herbal medicine and phytotherapy, a medicinal treatments based on plants or herbs and their extracts, have played a central role in the development of several anticancer agents such as Paclitaxel, Camptothecin, and Vincristine [7,8,9,10,11]. Herbal medicine can be utilized as an assistant therapeutic modality as well. It can enhance the response rate to chemotherapy as well as radiotherapy, help overcome resistance to therapy, decrease the severity of side effects caused by chemotherapy and radiotherapy, and enhance the quality of life and survival of cancer patients [12,13,14,15]. Herbal preparations and herbal active compounds act as anti-cancerous and anti-metastatic agents through several mechanisms, including scavenging of reactive oxygen species (ROS), modulation of epithelial-to-mesenchymal transition (EMT), impairment of angiogenesis, modification of the expression and activity of matrix metalloproteinases (MMPs), among others [16,17].

The Fabaceae family of plants includes a large number of domesticated species which are harvested as crops for human and animal consumption as well as oils, fuel, fertilizers, and medicinal and agricultural varieties [18]. Medicago is a genus of the Fabaceae family and it comprises more than 83–87 different species of flowering plants [18,19,20]. Twenty of these species are herbaceous perennials, sixty-three are herbaceous annuals, and only two are shrubs [20]. *Medicago orbicularis* L. Bartal. (common names: black disk, button medick, or button clover) is a species of the Medicago genus indigenous to Eurasia and North Africa [19]. The plant is distributed throughout the Mediterranean basin, mainly in Palestine, Lebanon, Syria, Algeria, Spain, France, Italy, and Greece, as well as in Middle Eastern countries such as Iraq and Iran, and several other countries [18]. *M. orbicularis* is a winter annual plant which flowers in spring and early summer. Its stems are leafy, its stipules are fimbriate, its leaflets are oval non-hairy with toothed margins, its flowers are orange-yellow, and its seed pods are flat, coiled, and lack spines [19].

*M. orbicularis’* phytochemical composition has not yet been investigated. But other Medicago species have been reported to be rich in phytochemicals, including flavonoids, carotenoids, saponins, phenolic acids, and phytoestrogens [21,22,23]. These phytochemicals have been demonstrated to exhibit anti-cancer activities through several mechanisms including apoptosis, antiproliferative, immunomodulatory, and anti-inflammatory activities, as well as their capacity to modulate oxidative stress [24,25,26,27,28]. In addition, close-by species such as *M. polymorpha*, *M. sativa*, *M. arabica*, or *M. truncatula* were shown to possess antioxidant activities [22,23]. A traditional therapeutic use of *M. orbicularis* was reported in Turkey for heart diseases [29]. However, there are currently no studies on the antioxidant potential, therapeutic applications, or anti-cancerous properties of *M. orbicularis*. Therefore, this study aimed to assess the antioxidant capacity, hemolytic properties, phytochemical composition, and the effects of *M. orbicularis* on cell proliferation and aggregation of A549 human lung cancer cells, as well as the combinatorial effect of cisplatin and *M. orbicularis* on A549 cells.

## 2. Materials and Methods

### 2.1. Cell Culture and Reagents

Human lung adenocarcinoma cell line A549 and primary normal neonatal fibroblast (HDFn) were obtained from the American Type Culture Collection (ATCC; Manassas, VA, USA). Cells were maintained in a humidified chamber at 37 °C and 5% CO_2_ in DMEM (Dulbecco’s Modified Eagle’s Medium; cat# D0819 Sigma-Aldrich Co., St. Louis, MO, USA) supplemented with 10% fetal bovine serum (FBS) (cat# F9665, Sigma-Aldrich) and 100 U/mL penicillin and 0.1 mg/mL streptomycin (cat# P4333, Sigma-Aldrich). Cells were passaged by trypsinization when they reached 90% confluence.

### 2.2. Plant Collection and Extraction

Fresh *Medicago orbicularis L. Bartal* was collected from Almat, Byblos, Lebanon between May and June 2021. A voucher specimen was stored in the herbarium of the Faculty of Pharmacy, Lebanese University, Beirut, Lebanon and was authenticated by Professor George Tohme, an experienced taxonomist and herbalist. *Medicago orbicularis L. Bartal:* Kingdom Plantae; Phylum Tracheophyta Class Magnoliopsida; Order Fabales; Family Fabaceae; Genus Medicago L. (Index Kewensis; https://powo.science.kew.org/taxon/urn:lsid:ipni.org:names:506322-1 (accessed on 20 October 2023)).

Extracts were obtained from leaves, stems, and fruits of *M. orbicularis*. In brief, the different plant parts were cleaned, air-dried in the shade at room temperature, ground to powder, and kept in plastic containers, away from light, heat, and moisture. Then, 10 g of the powder was mixed with 100 mL of 70% aqueous ethanol, and the mixture was placed in a reciprocating shaker and continuously agitated at 150 rpm for 3 to 4 days. The solutions were filtered and concentrated by a rotary evaporator at 40 °C and then lyophilized using a lyophilizer. The lyophilized extracts were stored at −20 °C until their use. At the time of use, the extracts were dissolved in 70% ethanol.

### 2.3. DPPH (α, α-Diphenyl-β-picrylhydrazyl) Antioxidant Activity

The antioxidant free radical scavenging ability of the ethanolic extracts of the different parts of *M. orbicularis* (leaves, fruits, and stems) was measured using the DPPH radical scavenging assay which was performed as described [30]. DPPH changes color from intense purple to pale-yellow due to the donation of a hydrogen atom, if the plant extract has radical scavenging activity. For *M. orbicularis* ethanolic extracts, 1 mL of different concentrations of the ethanolic plant extracts (50, 150, 250, 350, 500, 1000, 2500, and 4000 μg/mL) was mixed with an equal volume of DPPH (cat# D9312, Sigma-Aldrich Co.) solution (0.15 mM in ethanol). The blank consisted of 1 mL of DPPH solution and 1 mL of 70% ethanol. Mixed samples were then kept in the dark for 30 min and the OD was measured at a wavelength of 515 nm using a spectrophotometer. L-ascorbic acid, a potent antioxidant, was employed as a positive control and reference. The DPPH scavenging activity of each concentration of the extracts was calculated using the following formula:

$$\%Radical\;scavenging\;activity=\left[\frac{{OD}_{blank}-{OD}_{plant\;extract\;at\;each\;concentration}}{{OD}_{blank}}\right]\times 100$$

### 2.4. In Vitro Cytotoxicity Assay

In vitro cytotoxicity of *M. orbicularis* extracts was assessed using MTT assay, which is a reduction assay that measures cellular metabolic activity and is reflective of cell viability. A549 and neonatal fibroblast cells were seeded in a 96-well tissue culture plate at a density of 0.7 × 10^4^ cells per well in DMEM culture medium containing 10% FBS and penicillin/streptomycin. After an overnight incubation, cells were treated with different concentrations (50, 100, 150, 200, 300, or 500 μg/mL) of *M. orbicularis* extracts for 24, 48, and 72 h. As a control, cells were treated with a concentration of ethanol (vehicle-control cells) equal to the concentration of ethanol in the extract-treated cells. Following the treatment, MTT reagent (3-(4,5-Dimethylthiazol-2-yl)-2,5-Diphenyl Tetrazolium Bromide; cat# M5655, Sigma-Aldrich Co.) was added for 3 h until a purple formazan precipitate was formed. Media were removed gently, and an isopropanol-HCl solvent was added to dissolve the formazan precipitates. The optical density (OD) was measured by an ELISA plate reader at a wavelength of 595 nm. The percentage of cell viability of the treated cells was calculated using the following formula: $\%\;Cell\;Viability=(\frac{{OD}_{\;treated\;cells}\;}{{OD}_{vehicle-control\;cells}})\times 100$.

The IC50 (half-maximal inhibitory concentration) value was determined by extrapolation from the cell killing curve to determine the concentration of the extract that induced 50% cell death.

Cisplatin (Sigma-Aldrich Co.), dissolved in DMSO, was used as a positive control of the cytotoxic effect of *M. orbicularis’* parts’ extracts on A549 cells. A549 cells were treated for 24, 48, and 72 h with increasing concentrations of cisplatin (2.5, 5, 10, and 20 μg/mL) and compared to the DMSO-treated vehicle-control cells. IC50 values of cisplatin in A549 cells were determined at the three time points. Data are displayed as the mean ± SEM of three independent experiments.

### 2.5. Cell Aggregation Assay

Seeded A549 cells were detached using 2 mM EDTA PBS (calcium- and magnesium-free). Cells were pelleted and washed with PBS, and then resuspended in 1 mL PBS including or not 100 or 150 μg/mL of *M. orbicularis* fruit extract alone, 5 μg/mL cisplatin alone, or a combination of both and kept shaking on a rocker for 60 min at 37 °C in a cell culture incubator. Cells were fixed with 1 mL of 1% formaldehyde and imaged using an inverted microscope (Leica Microsystems GmbH, Wetzlar, Germany). Percentage of aggregation was calculated using the following formula: $\%\;Aggregation=(1-\frac{N_{\;t}}{N_{c}})\times 100$; where Nt is the number of single cells in treated wells and Nc is number of single cells in the vehicle-control cells.

### 2.6. Assay of Hemolysis and Anti-Hemolytic Activity

Hemolytic activity assay was performed as previously described [31]. Erythrocytes were obtained after separation from plasma by centrifugation at 2500 rpm for 10 min at 4 °C starting from fresh sheep blood. Briefly, RBCs were washed three times with 1X PBS and then pelleted and resuspended in PBS to obtain a 5% suspension of RBCs. Then, 50 μL of different concentrations (10, 20, 40, 50, and 100 μg/mL) of ethanolic extract of fruits of *M. orbicularis* were added to 1 mL of the RBC suspension. The mixture was incubated at 37 °C for 1.5 h; then, the suspension was centrifuged at 2500 rpm for 10 min at 4 °C and the OD measured at 540 nm using a spectrophotometer. Ethanol concentrations equal to those present in the fruit extracts were added to blood and used as a negative control, 1% SDS as a positive control of hemolysis of RBCs, and 1X PBS as the blank. Hemolytic levels were calculated as follows:

$$\%\;Hemolysis=(\frac{{OD}_{extract}\;\;}{{OD}_{\;vehicle-control}\;})\times 100$$

Anti-hemolytic activity assessment was performed as previously published [32]. Briefly, the same steps were performed as in the hemolysis assay except that H_2_O_2_ was added to induce hemolysis. After adding *M. orbicularis* extracts to the RBC suspension for 20 min, RBCs were incubated with 350 μL of 30% H_2_O_2_ at 37 °C for 1.5 h. The suspension was centrifuged at 2500 rpm for 10 min at 4 °C and OD at 540 nm was determined. A total of 30% H_2_O_2_ was used as a positive control, and 1X PBS as the blank. Anti-hemolytic levels were calculated according to the following equation:

$$\%\;inhibition\;of\;hemolysis=(\frac{{OD}_{vehicle-control}-{OD}_{extract}\;\;}{{OD}_{vehicle-control}\;})\times 100$$

### 2.7. Western Blotting Analysis

A549 cells were seeded in a 6-well plate at a density of 2.8 × 10^5^ cells per well and cultured for 24 h. The cells were treated with extracts of *M. orbicularis* for 48 h and then the cells were washed twice with PBS and lysed using a lysis buffer containing 2% SDS, 60 mM Tris lysis buffer (pH 6.8), and protease inhibitors and centrifuged at 10,000× *g* for 10 min. The protein concentration of the supernatants was determined using the Bradford protein assay kit (BioRad, Hercules, CA, USA) and 25 μg of protein lysates were resolved on 10% SDS-PAGE before being transferred to a polyvinylidene difluoride membrane (Immobilon PVDF; BioRad, Hercules, CA, USA). The membranes were then blocked for 1 h at room temperature with 5% non-fat dry milk in TBST (TBS and 0.05% Tween 20). Immunodetection was performed by incubating the membrane overnight with specific primary antibodies at 4 °C. Primary antibodies included rabbit anti-human B-cell lymphoma 2 (BCL-2) (Abcam, ab32124; dilution 1/5000), rabbit anti-Bcl-2 associated X protein (BAX) (Abcam, ab32503; dilution 1/2000), and rabbit anti-GAPDH (Abcam, ab181602; dilution 1/10,000). Membranes were washed and incubated with a horseradish peroxidase (HRP)-conjugated goat anti-rabbit IgG secondary antibody (Abcam, ab6721; dilution 1/5000) for 1 h followed by washing in TBST. Immunoreactive bands were detected using ECL substrate kit (Thermo Scientific, Rockford, IL, USA), and membranes were scanned using the Chemidoc imaging system (BioRad). The intensity of the obtained bands was quantified using ImageJ software version 1.53t (NIH, MD, USA). All bands were normalized to GAPDH, which was used as a loading control.

### 2.8. Spectroscopic Determination of Total Phenolic Content

Total phenolic compounds content (TPC) was determined using the Folin–Ciocalteu colorimetric oxidation/reduction-based reaction as previously described [33], with slight modifications. Briefly, 100 µL of diluted fruit extracts of *M. orbicularis* was added to 500 µL of Folin–Ciocalteu reagent, followed by the addition of 400 µL of saturated sodium carbonate solution (2%). The oxidation products of the reaction show a blue color with a broad light with an absorption maximum of 765 nm. OD 765 of the different mixtures was measured, against a blank, using a spectrophotometer. A calibration curve was prepared using a strong antioxidant, gallic acid, and the results were expressed as gallic acid equivalents (GAEs) in milligrams per gram of the extract.

### 2.9. Spectroscopic Determination of Total Flavonoids Content

The total flavonoids content (TFC) of *M. orbicularis* fruit extracts was determined by the aluminum chloride colorimetric method as previously detailed [34]. AlCl_3_ forms a flavonoid–aluminum complex that has a maximum OD at 510 nm. Briefly, a 100 µL aliquot of appropriately diluted fruit extracts of *M. orbicularis* was added to 400 µL of distilled water. Then, 400 µL of 5% NaNO_2_ was added, followed by 300 µL of 10% AlCl_3_. After 6 min of incubation 200 µL of 1 M NaOH was added to the mixture followed by 240 µL of distilled water. The absorbance of the resulting pink solution was determined at 510 nm versus a blank (water). A calibration curve was prepared using a known flavonoid, namely quercetin. The curve was used to calculate the amount of flavonoids in the plant extracts, which was expressed as quercetin equivalents in mg/g dry weight of the plant part.

### 2.10. Gas Chromatography/Mass Spectrometry (GC/MS)

The phytochemical compounds of *M. orbicularis* fruit extracts were analyzed using GC/MS analysis. A Perkin Elmer Clarus 680 (Perkin Elmer, USA) system attached to a triple quadrupole mass spectrometer was used. Chromatography was conducted on a hydrophobic capillary column RTxi-5 Sil MS column (30 m × 0.25 mm ID × 0.25 µm) using an injection volume of 10 µL, a flow rate of 1.5 mL/min, a pressure of 23.1 KPa, and an average velocity of 0.2 sec. The temperatures of the source and the interface were 200 °C and 280 °C, respectively. The initial temperature was set at 80 °C for 2 min, increased to 250 °C at 15 °C/min, and raised to 280 °C at 15 °C/min (held for 12 min). Identification of phytochemicals in extracts was carried out by comparing the obtained retention indices with those of chemical compounds in the database of the National Institute of Standards and Technology (NIST).

### 2.11. Statistical Analysis

Results were evaluated for statistical difference using one-way ANOVA followed by Bonferroni test to calculate *p* values. Data are presented as mean ± standard error of the mean (SEM) and a *p*-value of *p* < 0.05 was considered as statistically significant. Statistical analysis was performed using GraphPad Prism 8 software (GraphPad Software Inc., San Diego, CA, USA).

## 3. Results

### 3.1. Antioxidant Properties of M. orbicularis Plant Ethanolic Extracts

**Figure 1:**
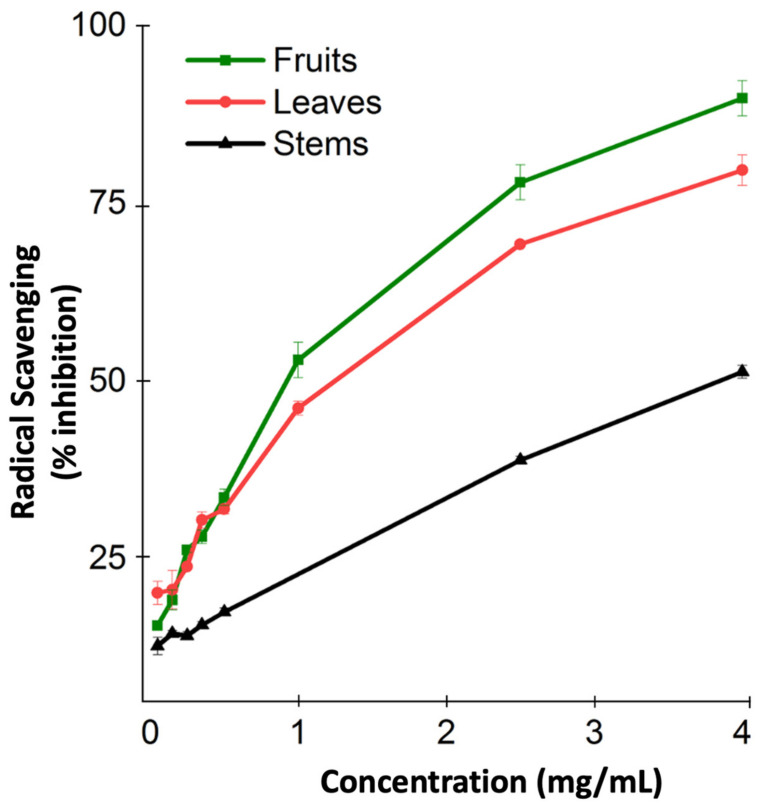
Ethanolic extracts of *M. orbicularis* exhibit antioxidant properties. The percentages of radical scavenging activity of different concentrations (50, 150, 250, 350, 500, 1000, 2500, and 4000 μg/mL) of leaves, fruits, and stems of *M. orbicularis’* ethanolic extracts were measured by DPPH radical scavenging assay. Data are presented as the mean ± SEM of three independent experiments (n = 3).

The antioxidant capacity of *M. orbicularis* was not measured before. Consequently, we used the DPPH radical scavenging assay to measure the antioxidant capacity of the different aerial parts of *M. orbicularis*. Figure 1 shows the antioxidant capacity of the ethanolic extracts of different parts of *M. orbicularis* extracts. The leaves’ ethanol extracts had the highest antioxidant potential, followed by the fruits’ ethanol extracts. *M. orbicularis’* stems’ ethanol extracts showed the lowest antioxidant activity. The calculation of the IC50 values confirmed these results (Table 1). The IC50 of radical scavenging activity of the strong antioxidant ascorbic acid is also shown in Table 1.

### 3.2. M. orbicularis’ Plant Parts’ Ethanolic Extracts Reduce the Viability of A549 Lung Adenocarcinoma Cells

**Figure 2:**
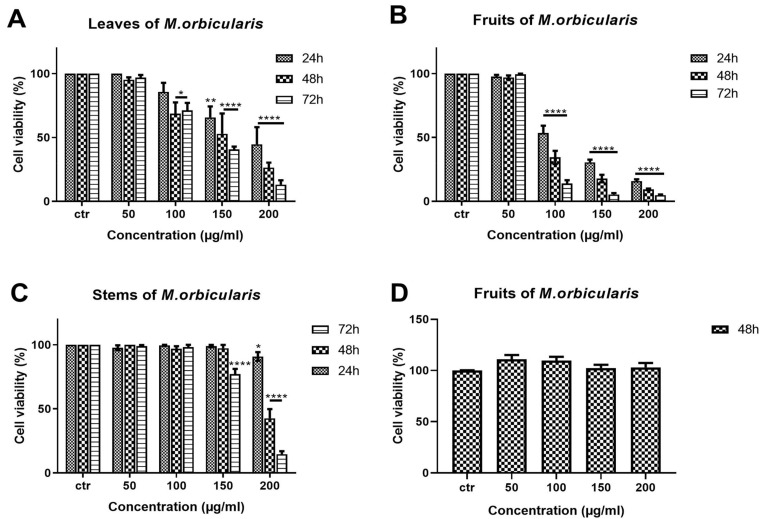
*M. orbicularis’* plant parts’ ethanolic extracts reduce the viability of A549 cells. MTT assay was used to measure cell viability of A549 cells treated for 24, 48, and 72 h with increasing concentrations (50, 100, 150, 200 μg/mL) of *M. orbicularis’* plant parts’ ethanolic extracts. (**A**–**C**) show the results of MTT cell cytotoxicity assay of A549 cells treated with the indicated concentrations of ethanolic plant extracts of *M. orbicularis* leaves, fruits, and stems, respectively. (**D**) shows the cell viability of neonatal fibroblast cells treated with the indicated concentrations of *M. orbicularis’* fruits’ ethanolic extract. Viability of treated cells was compared to the control-(ctr) vehicle-treated cells. Data are displayed as the mean ± SEM of three independent experiments (n = 3). * *p* < 0.05, ** *p* < 0.01, and **** *p* < 0.0001.

The anti-proliferative effects of different aerial parts of *M. orbicularis* extracts were tested against A549 human lung adenocarcinoma cells. A549 cells were treated with ethanolic extracts (50, 100, 150, 200 μg/mL) of *M. orbicularis* plant parts (leaves, fruits, and stems) for 24, 48, and 72 h, and their antiproliferative effects were determined using the metabolism-dye-based MTT assay (Figure 2). All plant extracts reduced the number of metabolically active A549 cells in a concentration- and time-dependent manner (Figure 2). *M. orbicularis’* leaves’ extracts at the concentration of 100 μg/mL caused a significant (*p* < 0.05) decrease in the number of A549 cells starting at 48 h, compared to the control-vehicle-treated cells (Figure 2A). *M. orbicularis’* fruits’ extracts, on the other hand, significantly (*p* < 0.0001) reduced the A549 cell number at the same starting concentration of 100 μg/mL, but at the earlier time point of 24 h (Figure 2B). Figure 2C shows that *M. orbicularis’* stems’ ethanolic extracts required a higher concentration (150 μg/mL) to cause a significant (*p* < 0.0001) decrease in the A549 cell number, at the later time point of 72 h. These data show that all *M. orbicularis’* parts’ ethanolic extracts can reduce the viability of A549 cells; *M. orbicularis’* fruits’ ethanolic extracts were the most potent at decreasing the viability of A549 cells. The antioxidant ability of *M. orbicularis* correlated with its anti-proliferative effects as previously reported for other plant extracts (Table 2) [30,35,36]. Table 2 shows that *M. orbicularis’* fruits’ ethanol extracts have the lowest IC50 value of A549 cell viability. The IC50 values of A549 cell viability were 116.5 ± 2.06 μg/mL, 91.86 ± 1.96 μg/mL, and 86.18 ± 1.93 μg/mL at 24, 48, and 72 h of treatment.

In addition, A549 cells were treated with the chemotherapeutic agent, cisplatin, which potently decreases A549 cell viability (Table 2). *M. orbicularis’* fruits’ ethanolic extracts show a decent inhibitory effect when compared with cisplatin (around 10 times higher IC50 at 48 h). Given that *M. orbicularis’* fruits’ ethanolic extracts were the most effective at reducing lung cancer cell viability, we decided to perform the next experiments using *M. orbicularis’* fruits’ ethanolic extracts.

The antiproliferative effects of *M. orbicularis’* ethanolic extracts are specific to cancer cells. *M. orbicularis’* fruits’ ethanolic extracts did not decrease the number of normal human neonatal fibroblast cells, even at the highest tested concentration of 200 μg/mL (Figure 2D). In comparison, the same concentration of fruit extracts decreased the number of A549 cells by 90% at 48 h, when compared to control-vehicle-treated cells (Figure 2B).

### 3.3. M. orbicularis’ Fruits’ Ethanolic Extracts May Induce Apoptosis of A549 Lung Adenocarcinoma Cells

**Figure 3:**
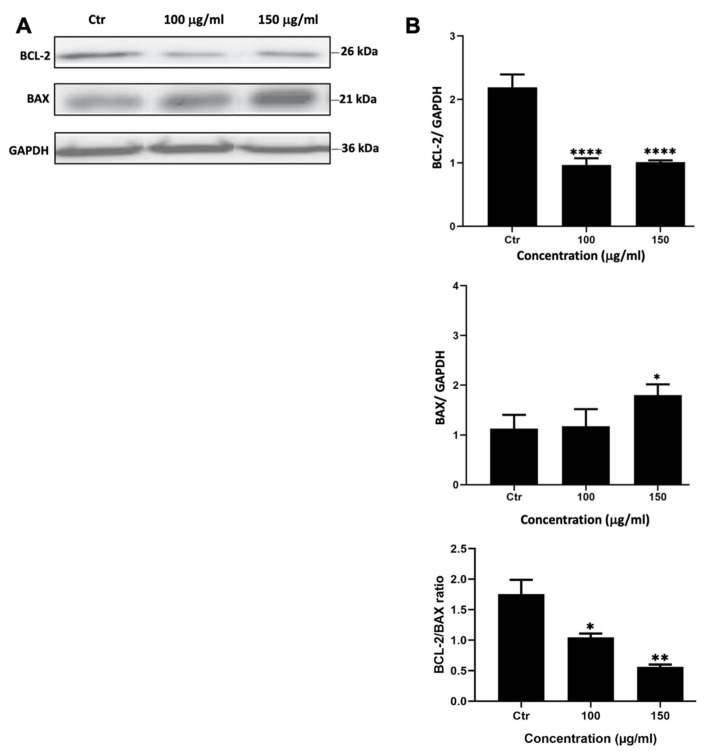
*M. orbicularis’* fruits’ ethanolic extracts induce apoptosis of A549 cells. (**A**) Representative Western blot of protein lysates from A549 cells treated 100 and 150 μg/mL of *M. orbicularis* fruit extracts for 48 h. Levels of proteins were normalized to GAPDH protein levels. (**B**) Quantification of images in (**A**). Intensity of protein bands were quantified by ImageJ software and normalized to intensity of bands of GAPDH protein. The ratio is expressed in arbitrary units. Data are presented as the mean ± SEM of 3 independent experiments (n = 3). * *p* < 0.05, ** *p* < 0.01, and **** *p* < 0.0001.

To assess whether *M. orbicularis* reduced the viability of A549 cells by inducing apoptosis, A549 cells were treated with *M. orbicularis* fruit extracts for 48 h. Protein lysates were subjected to Western blotting to evaluate the protein levels of the anti-apoptotic protein BCL-2 and the pro-apoptotic protein BAX, hallmark proteins of apoptosis. Figure 3 shows a significant decrease (*p* < 0.001) in the protein levels of BCL-2 in A549 cells treated with 100 and 150 μg/mL of *M. orbicularis* fruit extracts. BAX levels decreased after treating A549 cells with both 100 and 150 μg/mL of *M. orbicularis* fruit extracts, but the decrease was significant (*p* < 0.05) only at the concentration of 150 μg/mL (Figure 3B). The ratio of BCL-2/BAX, a ratio that determines the balance between anti- versus pro-apoptotic, was dose dependently and significantly (*p* < 0.05), decreased by treatment of A549 cells with 100 and 150 μg/mL of *M. orbicularis* fruit extracts. These results indicate that *M. orbicularis’* fruit extracts may induce apoptosis in A549 cells and that apoptosis may be responsible, at least in part, for *M. orbicularis*-induced cell death of A549 cells.

### 3.4. Ethanolic Extracts of M. orbicularis Fruits Exhibit Potent Antihemolytic Properties

**Figure 4:**
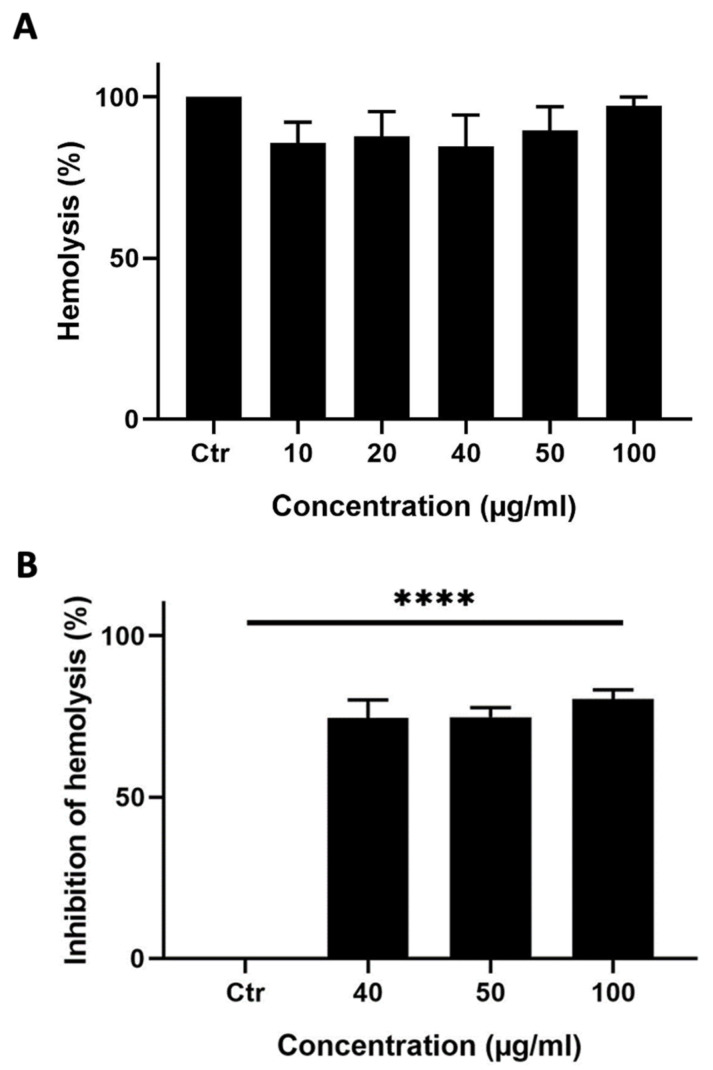
*M. orbicularis’* fruits’ ethanolic extracts show potent anti-hemolytic activity. (**A**) Hemolytic activity assay. Erythrocytes were treated with different concentrations (10, 20, 40, 50, and 100 μg/mL) of ethanolic extract of fruits of *M. orbicularis*. Bar graphs represent mean % hemolysis (compared to vehicle-control-treated RBCs) of 3 independent experiments. (**B**) Erythrocytes were pretreated with different (40, 50, and 100 μg/mL) concentrations of ethanolic extracts from the fruits of *M. orbicularis*. H_2_O_2_ was then added to induce hemolysis. Data represent % inhibition of hemolysis compared to control-(ctr) vehicle-treated cells. Data are displayed as the mean ± SEM of three independent experiments; **** denotes *p* < 0.0001.

A hemolysis assay was performed to test whether *M. orbicularis’* fruits’ ethanolic extracts can induce hemolysis of RBCs. Ethanolic extracts of *M. orbicularis* fruits did not cause hemolysis of RBCs (Figure 4A).

This result prompted us to test the ability of the ethanol extracts from the fruits of *M. orbicularis* to protect RBCs against hemolysis, which usually results from the use of chemotherapeutic agents in cancer therapy. A hemolysis assay was performed where RBCs were pretreated with different concentrations (40, 50, or 100 μg/mL) of the ethanolic extracts from the fruits of *M. orbicularis* and then treated with H_2_O_2_ to induce hemolysis (Figure 4B). Figure 4B shows that 40, 50, or 100 μg/mL of ethanolic extracts of *M. orbicularis* fruits significantly inhibited hemolysis of RBCs. For example, a concentration of 100 μg/mL of fruit extracts of *M. orbicularis* protected 75.65% of RBCs from hemolysis. This result sheds light on the safety of fruit extracts of *M. orbicularis* and underscores the potential use of these extracts in combination therapy with classical therapeutic agents, known to induce RBC hemolysis.

### 3.5. Ethanolic Extracts of Fruits of M. orbicularis Enhance Cisplatin-Induced Cytotoxicity in A549 Lung Cancer Cells

Cisplatin has potent anti-proliferative effects against A549 cells [37], and the anti-proliferative effect of cisplatin was confirmed in this study. The IC50 values of the cisplatin-induced reduction in viability of A549 cells were 24.73, 9.56, and 4.72 μg/mL at 24, 48, and 72 h, respectively (Table 1). These values agree with the previously published literature [37,38].

**Figure 5:**
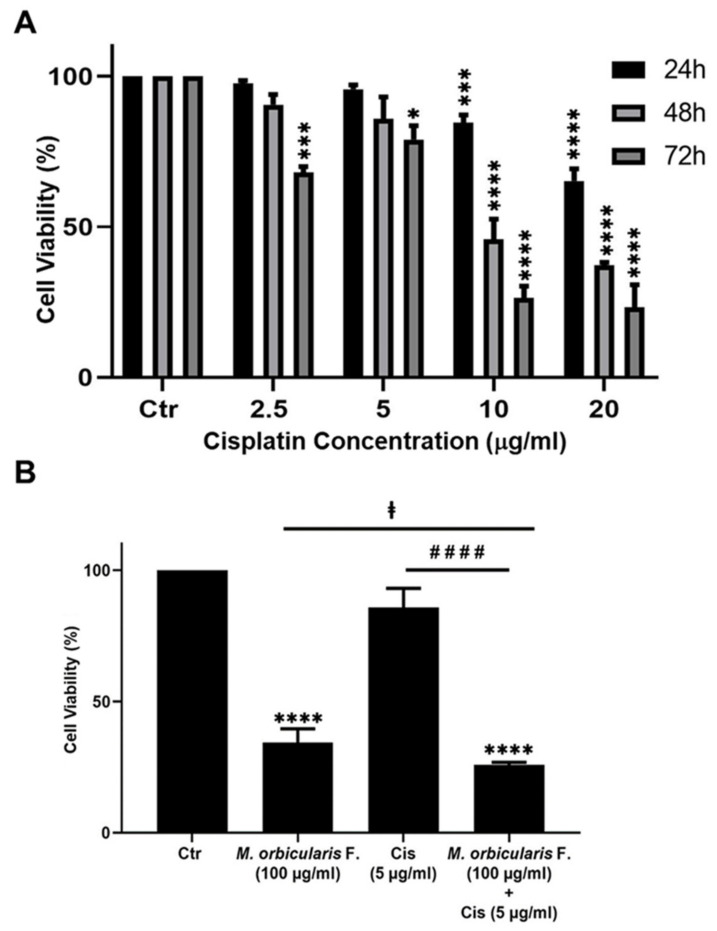
*M. orbicularis* enhance cisplatin-induced cytotoxicity in A549 lung cancer cells. (**A**) Cell viability of A549 cells treated with the indicated concentrations of cisplatin was measured using MTT assay. Data are displayed as % cell viability relative to control (ctr) cells. * *p* < 0.05, *** *p* < 0.001, and **** *p* < 0.0001 (**B**) A549 cells were treated with fruit extracts of *M. orbicularis* (100 μg/mL) alone or combined with 5 μg/mL cisplatin (Cis) for 48 h, and then assayed for cell viability using MTT assay. Bar graphs represent % cell viability relative to control (ctr) cells. Data are presented as the mean ± SEM of three independent experiments (n = 3). Significant difference from control: **** *p* < 0.0001; significant difference from cisplatin treatment alone: #### denotes *p* < 0.0001; and significant difference from *M. orbicularis* alone: ‡ denotes *p* < 0.05. *M. orbicularis* F. denotes *M. orbicularis* fruits extracts.

Many types of cancer, including lung cancer, develop resistance to chemotherapy and, as a result, they usually cause relapse following chemotherapy with cisplatin or other chemotherapeutic agents [39]. Combination therapy can help to overcome the chemoresistance that develops against chemotherapeutic agents. When combined with chemotherapy, plant extracts with anti-cancerous properties may offer a way of overcoming chemoresistance and cancer relapse. This is because plant preparations usually have a different mechanism of action than chemotherapeutic agents [16]. Therefore, we assessed the effect of a combination treatment using ethanolic extracts of *M. orbicularis* fruits on cisplatin-induced death of A549 cells. A549 cells were treated for 48 h with a low-dose of cisplatin (5 μg/mL) in combination with 100 μg/mL of *M. orbicularis’* fruits’ extracts. Cell viability assays showed that, at these concentrations of cisplatin alone or *M. orbicularis’* fruits’ extracts alone, there were moderate cytotoxicity levels (Table 1, Figure 2B and Figure 5A). In the combination treatment, the cell viability of A549 decreased significantly (*p* < 0.0001) from 85.9% in A549 cells treated with cisplatin alone to 25.8% in cells treated with the combination of cisplatin and *M. orbicularis’* fruits’ extracts (Figure 5B). In addition, the combination treatment enhanced the cytotoxic effect of *M. orbicularis’* fruits’ extracts where A549 cell viability decreased from 34.4% in cells treated with *M. orbicularis’* fruits’ extracts alone to 25.8% in the combination treatment (Figure 5B). These results show that *M. orbicularis’* fruits’ ethanolic extracts sensitized A549 cells to the cytotoxic effects of cisplatin.

### 3.6. Ethanolic Extracts of Fruits of M. orbicularis Augment Cisplatin-Induced Aggregation of A549 Lung Cancer Cells

**Figure 6:**
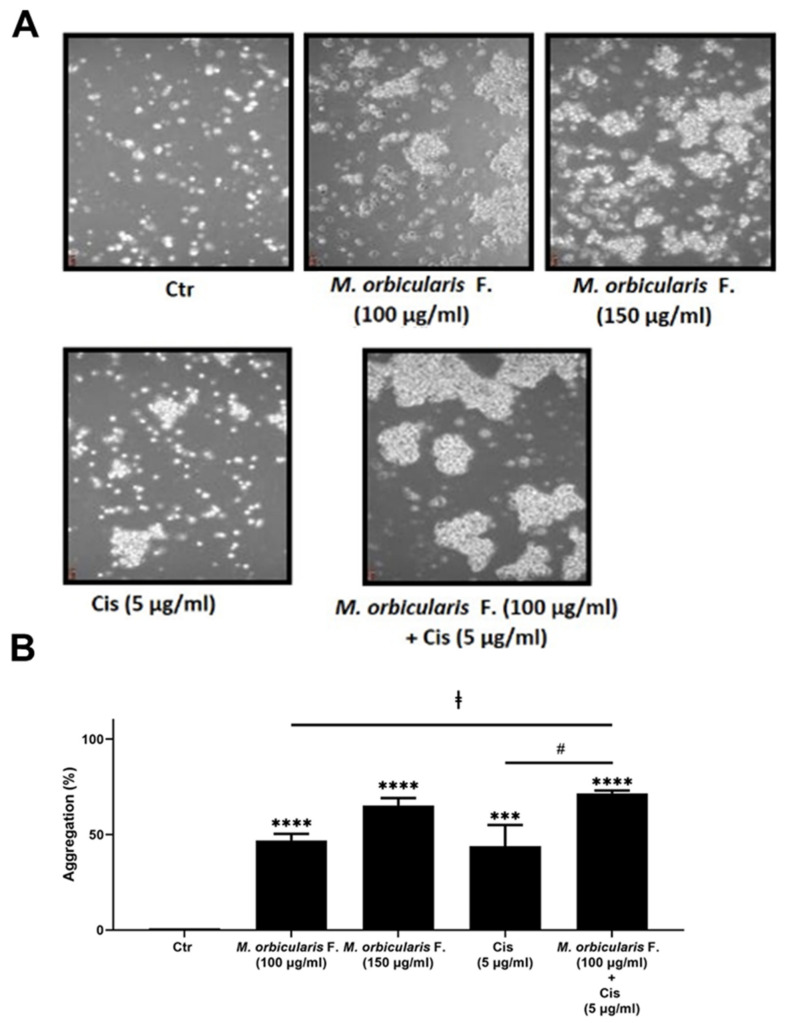
*M. orbicularis* augments cisplatin-induced aggregation of A549 lung cancer cells. (**A**) A549 cells were incubated with *M. orbicularis* alone (100 or 150 μg/mL) or combined with 5 μg/mL cisplatin (Cis), and then subjected to cell aggregation assay as described in the Materials and Methods Section. (**B**) Quantification of the data in (**A**). Micrographs of cells were taken after 1 h of treatment and the percentage of cell–cell aggregations was measured using the following equation: % aggregation = (1 − Nt/Nc) × 100, where Nt is the number of single cells in the control and Nc is the number of single cells in the treated sample. Data represent the mean ± SEM of three independent experiments (n = 3). Significant difference from control: *** *p* < 0.001, and **** *p* < 0.0001; significant difference from cisplatin treatment alone: # denotes *p* < 0.005; and significant difference from *M. orbicularis* extracts alone: ‡ denotes *p* < 0.05. *M. orbicularis* F. denotes *M. orbicularis’* fruits’ extracts.

We next sought to examine the effect of *M. orbicularis* fruit extracts on the cell–cell adhesion of A549 cells using a cell aggregation assay. Figure 6 shows that, after 1 h of treatment of A549 cells with 100 and 150 μg/mL of *M. orbicularis* fruit extracts, the percentages of cell aggregation significantly (*p* < 0.001 in comparison to vehicle control cells) increased by 47% and 65%, respectively (Figure 6B). The percentage of cell aggregation of A549 is 44% after treatment with 5 μg/mL cisplatin alone and significantly increased to 71.5% after the co-treatment with cisplatin (5 μg/mL) and *M. orbicularis* (150 μg/mL) (Figure 6A,B). These results suggest that the *M. orbicularis* extract significantly augmented the cisplatin-induced aggregation of A549 lung cancer cells.

### 3.7. Total Polyphenol and Flavonoid Contents of M. orbicularis’ Fruits’ Ethanolic Extracts

Since the phytochemicals of *M. orbicularis* have not been previously identified, the total phenolic and flavonoid contents of *M. orbicularis’* fruits’ extracts were assayed. The total phenolics content was 1.1583 ± 0.00005 mg GAE/g of dry matter of *Medicago orbicularis* fruits. The total flavonoids content was 0.0246 ± 0.00003 mg quercetin equivalents/g of dry matter of *Medicago orbicularis* fruits. These results show that *Medicago orbicularis* fruits are rich in phenolics and flavonoids and may explain the anti-cancer properties of *Medicago orbicularis* fruits against A549 cells. Anti-cancerous properties of plant extracts are often attributed to polyphenols and flavonoids [22,26,40,41,42].

### 3.8. Identification of Phytochemical Composition of M. orbicularis’ Fruits’ Ethanolic Extracts by GC/MS

**Figure 7:**
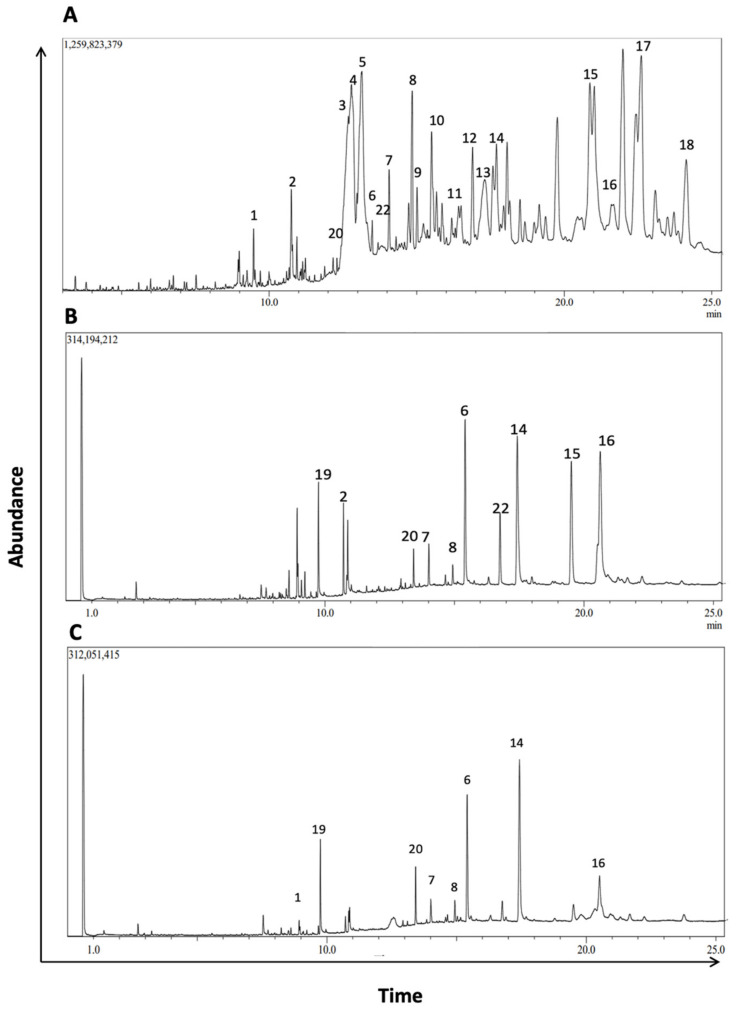
GC/MS chromatogram of *M. orbicularis’* parts’ ethanolic extract displaying the elution profile of phytochemicals listed in Table 3. (**A**) Fruit extracts, (**B**) leaf extracts, and (**C**) stem extracts.

To further characterize the phytochemical contents of *M. orbicularis* fruits, GC/MS analysis was used (Figure 7). Table 2 lists 18 of the major compounds of *M. orbicularis* fruits as identified by GC/MS, by comparing their mass spectral fragmentation patterns to those of known compounds listed in the NIST library.

## 4. Discussion

Cancer is a complex and devastating disease which affects millions of lives worldwide. Lung cancer, particularly non-small cell lung cancer, has the highest mortality rate among malignant tumors across the globe [37]. Current treatments for lung cancer such as chemotherapy and radiotherapy are associated with considerable toxicity and other side effects. In addition, lung cancer patients develop resistance to therapy and suffer from lung cancer recurrence as early as 6 months following therapy [3,4]. In this regard, there is a revived research interest in using herbal- and plant-based therapies as sources of anti-cancer bioactives. Several herbal remedies have been shown to have efficacy as well as minimal toxicity and side effects for disease treatment [12,13]. In addition, plants and their phytochemicals are being used as starting scaffolds for developing more potent anti-cancer agents [15,43]. Historically, plants or herbs and their extracts were used for the development of several important anti-cancer agents such as Paclitaxel, Camptothecin, and Vincristine [7,8,9,10,11,43]. Furthermore, several intricate biochemical pathways are simultaneously dysregulated in cancer [6,15], and herbal-based or herbal-derived remedies can target several molecular pathways and can become an alternative or complementary treatment to conventional cancer therapies [7,12,13,14,15,44,45]. Relatedly, multiple recent in vitro and in vivo studies demonstrated that the concurrent use of natural products with conventional treatments (chemo- and radiotherapy) can synergistically sensitize tumors to therapy, enhancing therapeutic efficacy and reducing toxicities [15]. Many studies have documented the promising use of herbal plant extracts against cancer cell lines in vitro and in vivo in animal models of cancer and are now being tested in clinical trials. These studies have shown that herbal-based remedies have antioxidant, antihemolytic, and apoptosis-inducing effects. They can also impact cell proliferation, aggregation and adhesion, migration, and metastasis [16,17,46].

*Medicago orbicularis* L. Bartal is an understudied plant species as far as its therapeutic effects are concerned. Except for a study reporting a traditional use of *M. orbicularis* for heart diseases management in Turkey [29], there are no reports on the therapeutic applications or phytochemical composition of *M. orbicularis*. Notably, *M. orbicularis’* anti-cancerous activities have not been explored yet [18]. It was therefore pertinent to study the phytochemical composition, antioxidant capacity, antihemolytic properties, and the effects of *M. orbicularis* on cell proliferation, apoptosis, and cell aggregation potential against human lung adenocarcinoma A549 cells, in addition to the combinatorial effect of a treatment of both the chemotherapeutic agent cisplatin and *M. orbicularis* on A549 cells.

There are no studies currently on the anti-oxidant potential of *M. orbicularis*, but other species of the genus Medicago such as *M. polymorpha*, *M. sativa*, *M. arabica*, and *M. truncatula* were shown to have anti-oxidant activities and to be rich in phytochemicals [22,23]. Since anti-oxidant activity is often accompanied by therapeutic effects [22,23,27,28,43,47,48,49,50], this study was initiated with an evaluation of the antioxidant activity of the ethanolic extract of different plant parts of *M. orbicularis,* including its leaves, fruits and stems. All plant parts of *M. orbicularis* showed modest anti-oxidant potential and the leaves showed the highest antioxidant activity.

All *M. orbicularis* plant parts inhibited the proliferation of A549 cells, but *M. orbicularis* fruits exhibited the highest reduction in viability of A549 cells and were chosen to perform the rest of the experiments of the study. The National Cancer Institute (NCI, USA) considers an IC50 of 30 μg/mL to indicate a strong cytotoxic activity and a promising candidate for the further purification of a crude extract, and an IC50 of 31–200 μg/mL to indicate moderate cytotoxicity [51]. The IC50 of the fruit extracts at 72 h of treatment was 86.18 ± 1.93 μg/mL, indicating that this crude extract has moderate cytotoxicity. To date, no other study has reported the cytotoxicity levels of *M. orbicularis* against cancerous cells. Importantly, the cytotoxic effects of the fruit extracts showed selectivity to A549 cancerous cells and did not affect human neonatal fibroblast cells even at high concentrations. This indicated that *M. orbicularis’* fruits’ extracts may have no side effects when used in vivo.

Plant bioactives can decrease the viability of cancer cells through several mechanisms, including the induction of apoptosis [7,12,13,14,15,44,45]. Cytotoxic agents can cause an increase in the expression of the pro-apoptotic protein BAX that makes pores in the mitochondrial membrane, leading to the release of cytochrome C [52]. The release of cytochrome C initiates the execution phase of apoptosis and activates Caspase 9, which cleaves pro-Caspase 3 into active Caspase 3, the main executer effector caspase [53,54]. Active Caspase 3 can cleave many protein substrates [55], such as caspase-activated DNAse that fragments genomic DNA [56] and poly-adenosine diphosphate (ADP) ribose polymerase-1 (PARP-1), to promote apoptosis [54]. These events are recognized as the intrinsic apoptotic pathway. The anti-apoptotic protein BCL-2 can inhibit the release of cytochrome C into the cytoplasm, thereby attenuating the intrinsic apoptotic pathway [54,57]. Western blotting analysis of lysates of A549 cells treated with 100 and 150 μg/mL *M. orbicularis* fruit extract indicated that *M. orbicularis* fruit extracts lowered the expression of BCL-2 and increased the expression of BAX, suggesting the activation of the intrinsic apoptotic pathway. Consequently, there was a dose-dependent decrease in the BCL-2/BAX ratio. This is the first report showing that *M. orbicularis*-induced death of A549 cells may be mediated, at least partly, by the intrinsic apoptotic machinery. However, confirmation of the induction of the intrinsic apoptosis pathway is warranted in future investigations, particularly the activation of Caspase 3 and PARP-1. Other mechanisms of cell death, such as autophagy, necroptosis, or pyroptosis, may also be induced by *M. orbicularis*, and need to be tested in future studies.

As part of the safety profile of a cancer drug, its systemic infusion through the blood of a patient should not cause any hemolysis of red blood cells [58]. *M. orbicularis* fruit extracts did not cause any hemolysis of RBCs. Moreover, the fruits extracts protected RBCs against hemolysis. This attested to the possible safety of these extracts in future in vivo or clinical studies.

Metastasis is the major culprit behind cancer-associated mortality [59,60,61]. For cancer cells to metastasize, they should lose their adhesion to neighboring cells, allowing them to migrate and invade at secondary tumor sites and organs [27,28,60,61,62]. Cancer therapeutics can act by strengthening cell adhesion and aggregation to prevent cell migration and metastasis [63]. In this study, an aggregation assay showed that *M. orbicularis* fruits significantly enhanced cell aggregation, signifying that the extract enhanced the adhesion of A549 cells. This result attests to the ability of *M. orbicularis* to attenuate the malignant phenotype of A549 cells. Future studies should focus on elucidating the molecular mechanisms underlying this finding, including the examination of protein kinases involved in cell adhesion and migration such as Jun N-terminal kinase (JNK) and p38 mitogen-activated protein kinases, cell adhesion proteins, connexins gap junction proteins, and EMT markers such as E-cadherin, N-cadherin, vimentin, and Snail, among others [62,64].

Apart from their direct suppressive effects on carcinogenesis and cancer metastasis, herbal remedies and plant extracts have been used to complement conventional therapies since they can target several molecular pathways other than those targeted by conventional therapy, and therefore may overcome the refractoriness that develops against cancer therapeutics [7,12,13,14,15,44,45]. When combined with chemotherapy, plant-based therapies can enhance the response to chemotherapy, help overcome resistance to therapy, and decrease the severity of the side effects of chemotherapy, including cancer-related fatigue [7,12,13,14,15]. Chemotherapeutic agents, including cisplatin, can become refractory in lung cancer patients, and as result the cancer may relapse with poor prognosis [3,4]. The combination of cisplatin with a multitargeted herbal remedy may circumvent this problem. In this study, the cytotoxic effect of cisplatin alone on A549 cells was confirmed. The IC50 values of cisplatin with A549 that we obtained are similar to those reported in the literature [37]. In order to determine the impact of a combination treatment on the cisplatin-induced death of A549 cells, we applied a co-treatment of cisplatin and the ethanolic extract of *M. orbicularis* fruits. The results show that the combination treatment significantly augmented the cisplatin-induced decrease in the cell viability of A549 cells, when compared to either the extract alone or cisplatin alone. These results show that *M. orbicularis* may be a source of agents for a complementary therapy for lung cancer, in combination with chemotherapy. The combination treatment showed a similar result when tested on an aggregation of A549 cells. The co-treatment of *M. orbicularis’* fruits’ extracts and cisplatin proved to significantly augment the increase in aggregation of A549 cells, when compared to either the extract alone or cisplatin alone. Taken together, these results support *M. orbicularis* as a source for the development of anti-lung cancer drug candidates that can complement chemotherapy.

*M. orbicularis’* phytochemical composition has not been defined yet. But other Medicago species have been reported to be rich in phytochemicals [21,22,23], which have anti-cancer activities [24,25,26,27,28]. Natural polyphenols are plant secondary metabolites which have two or more phenol rings. Polyphenols’ health benefits include being antioxidant, antidiabetic, cardioprotective, and neuroprotective. Relatedly, phenolics have been reported to have strong anti-cancer effects through various mechanisms including removal of cancer cells, inhibition of cell cycle, induction of apoptosis, and inhibition of metastasis, among others [42]. Flavonoids are natural polyphenols with documented anti-cancer properties. Flavonoids have been reported to inhibit carcinogenesis by suppressing oxidative stress through their antioxidant activities [26,41]. Our results indicate that *M. orbicularis’* fruits’ ethanolic extracts have cytotoxic effects against A549 cells. These effects could be mediated by polyphenols and flavonoids of *M. orbicularis’* fruits’ extracts among other phytochemicals. In this study, *Medicago orbicularis* fruits were shown to be rich in phenolics and flavonoids, which may explain the anti-oxidant and anti-cancer properties of *Medicago orbicularis* fruits against A549 cells.

To further evaluate the phytochemical composition and establish a more comprehensive profile of the phytochemical constituents of *M. orbicularis* fruits, GC/MS analysis was conducted. We identified 20 prominent peaks that correspond to bioactive compounds of *M. orbicularis* fruits. The *M. orbicularis’* fruits’ ethanol extract was found to be a complex mixture of various classes of phytochemicals, including flavonoids, alkaloids, diterpenes, triterpenes, sesquiterpenes, sterols, alcohols, aldehydes, and fatty acids. Among the diverse array of phytochemicals present in the extract, several compounds were correlated with therapeutic properties, including anti-cancerous activities. For example, lupeol and its derivatives, which were the most frequently identified compounds, have been recently demonstrated to possess a diverse array of pharmacological activities [65]. These activities included anticancer, antimicrobial, and antidiabetic effects, with certain lupeol derivatives exhibiting greater potency than lupeol [66]. Furthermore, the phytochemical composition of the stems and leaves of *M. orbicularis* was also identified by GC/MS. This revealed that *M. orbicularis* fruits have more phytochemical types than either the leaves or the stems. Moreover, a comparison of the abundance of the phytochemicals commonly present in fruits, leaves, and stems may provide hints about the phytochemicals responsible for the antiproliferative activities of *M. orbicularis’* fruits’ extracts. For example, lupeol and its derivatives have anti-cancerous activities [65,66], and are more enriched in the fruits’ extracts. Future investigation should focus on a more comprehensive assessment of the plant extracts of *M. orbicularis*, using more advanced techniques such as preparative HPLC followed by LC-MS/MS or NMR, to identify the bioactives responsible for the therapeutic effects of crude extracts from this plant.

In conclusion, this study has determined, for the first time, the anti-oxidant, anti-hemolytic, and cytotoxic properties of the hydroalcoholic extracts of *M. orbicularis* against A549 lung cancer cells. The decrease in cell viability may be, at least partly, mediated by the intrinsic pathway of apoptosis, pending confirmation by future studies. *M. orbicularis* was also able to enhance the cell aggregation of A549 cells. These activities could be attributed to the phytochemicals present in *M. orbicularis* such as polyphenols and flavonoids, and the other compounds that were identified by GC/MS. These properties place *M. orbicularis* as a potential new candidate to offer effective natural agents for the treatment of lung cancer.

## Figures and Tables

**Table 1:** Antioxidant activity of ethanolic extracts of *M. orbicularis* parts.

Plant Part	IC50 mg/mL (Radical Scavenging Activity)
*M. orbicularis* Fruits	0.907 ± 0.75
*M. orbicularis* Leaves	1.993 ± 0.6
*M. orbicularis* Stems	3.732 ± 1.02
L. Ascorbic acid	0.0275 ± 1.32

**Table 2:** IC50 values (in μg/mL) of ethanolic extracts of parts of *M. orbicularis* used to treat A549 cells for 24, 48, and 72 h. The IC50 values of cisplatin in A549 cells are also listed for comparison.

A549 Cells			
	24 h	48 h	72 h
*M. orbicularis* Leaves	146.8 ± 2.16	144.6 ± 1.16	130 ± 2.11
*M. orbicularis* Fruits	116.5 ± 2.06	91.86 ± 1.96	86.18 ± 1.93
*M. orbicularis* Stems	310 ± 2.49	194 ± 2.29	177 ± 2.25
Cisplatin	24.73 ± 1.46	9.56 ± 1.07	4.71 ± 0.88

**Table 3:** Results of GC-MS analysis of extracts of *M. orbicularis* fruits, leaves, and stems. The table presents the name of the identified compounds and their relative abundance in the plant parts. The listed compounds have highest similarity indices to candidates in the NIST database.

S. No	Compound Name	Compound Nature	Leaves	Stems	Fruits
1	7-Hexadecenal, (Z)	Unsaturated aldehyde	+	+	++
2	cis-9,cis-12-Octadecadienoic acid	Unsaturated fatty acid	++	+	++
3	Lup-20(29)-en-3-ol, acetate, (3 beta)	Triterpenes	+	+	+++
4	Acetyllithocholic acid, methyl ester	Lithocholic acid	—	—	+++
5	12-Oleanen-3-yl acetate, (3 alpha)	Triterpenoids	—	—	+++
6	n-Pentadecanol	Alcohol	+++	+++	++
7	2-Pentacosanone	Fatty ketone	++	++	+++
8	Oxirane, hexadecyl-	Cetyl epoxide	++	++	+++
9	4-Nitrophenyl laurate	4-Nitrophenyl esters	—	—	+++
10	Lupeol, trifluoroacetate	Triterpene alcohol	—	+	+++
11	N-Methyl-pseudotomatidine diacetate	Alkaloid	+++	—	+++
12	Pyridine, 1-acetyl-1,2,3,4-tetrahydro-5-(2-piperidinyl)	Piperidine alkaloid	—	—	++
13	2-Bornanone oxime	Bicyclic terpene	—	+	++
14	dl-alpha-Tocopherol	Vitamin fat soluble	+++	+++	++
15	gamma-Sitosterol	Sterol	+	+	++
16	beta-Amyrone	Sesquiterpene	+++	+	++++
17	Lupeol	Triterpene	+	+	+++
18	Dihydroniloticin diacetate	Flavonoids	+	+	+++
19	n-Hexadecanoic acid	Fatty acid	+	+	—
20	Apigenin	Flavone	++	++	—

+: present; ++: abundant; +++ highly abundant; —: absent.

## Data Availability

Data is contained within the article.
